# Task-dependent pitch auditory feedback control in cerebellar ataxia

**DOI:** 10.21203/rs.3.rs-3186155/v1

**Published:** 2023-07-25

**Authors:** Allison Hilger, Jennifer Cole, Chuck Larson

**Affiliations:** University of Colorado Boulder; Northwestern University; Northwestern University

**Keywords:** ataxia, dysarthria, cerebellum, speech language pathology, pitch, voice

## Abstract

**Purpose:**

The purpose of this study was to investigate how ataxia affects the task-dependent role of pitch auditory feedback control in speech. In previous research, individuals with ataxia produced over-corrected, hypermetric compensatory responses to unexpected pitch and formant frequency perturbations in auditory feedback in sustained vowels and single words (Houde et al., 2019; Li et al., 2019; Parrell et al., 2017). In this study, we investigated whether ataxia would also affect the task-dependent role of the auditory feedback control system, measuring whether pitch-shift responses would be mediated by speech task or semantic focus pattern as they are in neurologically healthy speakers.

**Methods:**

Twenty-two adults with ataxia and 29 age- and sex-matched control participants produced sustained vowels and sentences with and without corrective focus while their auditory feedback was briefly and unexpectedly perturbed in pitch by +/−200 cents. The magnitude and latency of the reflexive pitch-shift responses were measured as a reflection of auditory feedback control.

**Results:**

Individuals with ataxia produced larger reflexive pitch-shift responses in both the sustained-vowel and sentence-production tasks than the control participants. Additionally, a differential response magnitude was observed by task and sentence focus pattern for both groups.

**Conclusion:**

These findings demonstrate that even though accuracy of auditory feedback control correction is affected by cerebellar damage, as evidenced by the hypermetric responses, the system still retains efficiency in utilizing the task-dependent role of auditory feedback.

## Introduction

Ataxia is a neurological condition that results from damage to the cerebellum, affecting movement coordination across the body (Diener & Dichgans, 1992; Manto & Marmolino, 2009). Speech, a highly coordinative activity, is uniquely impacted in ataxia (Kent et al., 2000). Individuals with ataxia exhibit significant impairment in the naturalness of speech (i.e., pitch, loudness and timing) but intelligibility (i.e., how well words can be understood) is often spared or minimally affected, suggesting differential deficits in neural control for speech (Hilger et al., 2022). The goal of this study was to assess neural control of speech in ataxia, specifically for feedback control of pitch.

A possible reason that speech naturalness is more disrupted than speech intelligibility in ataxia is because feedback control is impaired to a greater extent than feedforward control. Speech naturalness is strongly correlated with prosodic aspects of speech (i.e., fundamental frequency (*f*_*o*_), vocal intensity, and timing), and is heavily reliant on feedback control (Guenther, 2016; Perkell et al., 2007; Yorkston et al., 1999). Feedback control is important for prosody to make salient distinctions in *relative acoustic prominence*, or the relative enhancement of phrasal stress within a phrase (Aylett & Turk, 2004; Cole, 2015; Kochanski et al., 2005; Tamburini & Caini, 2005; Turk & White, 1999). Adjusting the subtle, relative acoustic features of speech within the production of a phrase requires careful and precise self-monitoring of speech, which is known as auditory feedback control (Guenther, 2016; Tourville & Guenther, 2011). In this study, we investigate feedback control in ataxia to better understand the role of the cerebellum in pitch control and prosody. We hypothesize that due to cerebellar damage, feedback control is impaired in the adjustment of auditory targets based on sensory prediction and in generating corrective commands in response to sensory errors.

### a. The role of the cerebellum in feedback control of speech

The cerebellum plays an important role in predicting the sensory consequences of actions, which has important implications for adjusting production targets for speech (Davidson & Wolpert, 2005; Desmurget & Grafton, 2000; Guenther, 2016; Kawato, 1999; Larson et al., 1978; Miall & Wolpert, 1996). During speech production, cortical mechanisms send motor plans forward for production by generating auditory and somatosensory targets that are then mapped onto articulatory gestures for production (Guenther, 2016; Guenther et al., 2006; Tourville & Guenther, 2011). These targets are also used in ongoing production to compare incoming feedback with the intended targets to identify errors through mismatches in these comparisons (Tourville & Guenther, 2011).

The cerebellum provides indirect mediation in the generation of the auditory target (Guenther, 2016). The auditory target is a learned motor program for a speech movement, and is adjusted by the cerebellum for production based on the current gestural state and communicative context (Tourville & Guenther, 2011). For example, the intended loudness of the auditory target may be adjusted depending on whether the speaker is in a quiet library or a loud restaurant. When the cerebellum is damaged, it is likely that it is unable to accurately estimate the necessary adjustments for the auditory target based on sensory information. It is possible that this damage creates an inaccurate estimation of the auditory target depending on the complexity of the production target and the extent of cerebellar damage.

We theorize that this inaccurate adjustment of the auditory target has implications for feedback control in speech. The generation of production targets, such as auditory targets, is typically viewed mainly as a feedforward process that sends motor programs forward for production (Guenther, 2016; Tourville & Guenther, 2011). Feedforward impairments are observed in ataxia, providing evidence for a cerebellar role in feedforward control of auditory targets for speech (Parrell et al., 2017). However, we theorize that inaccurate adjustments of the auditory target also help account for feedback control impairments to correct for errors. As described above, the cerebellum adjusts the auditory target based on sensory prediction, and that auditory target is then used for production mechanisms. However, that same auditory target is also copied to feedback control systems to compare incoming auditory feedback with the intended auditory target (Tourville & Guenther, 2011). If the auditory target is incorrectly estimated by the cerebellum, then comparisons with incoming feedback will also be incorrectly estimated. The result is that more errors, and greater errors, may be detected in ongoing speech, triggering feedback control to make more corrections.

However, there is an additional feedback process that is impaired in ataxia. The cerebellum not only makes adjustments to the auditory target but also to the corrective movements that are generated when errors are identified in speech (Diedrichsen et al., 2005; Grafton et al., 2008; Penhune & Doyon, 2005; Rondi-Reig et al., 2014). More specifically, the corrective movements are indirectly influenced by sensory error representations in the cerebellum (Guenther, 2016). If the cerebellum is damaged, then the cerebellar adjustments made to the corrective movements will also be inaccurate. Therefore, auditory feedback control is considerably impaired in ataxia because of disruption to the cerebellum’s roles in adjusting both the auditory target for comparisons to incoming feedback as well as the corrective movements when errors are identified from these comparisons.

This hypothesis is supported by recent studies that have shown hypermetric reflexive responses to auditory feedback perturbations in pitch and vowel formant frequencies (Houde et al., 2019; Li et al., 2019; Parrell et al., 2017). The auditory feedback perturbation paradigm is experimentally utilized to assess the efficiency and accuracy of the auditory feedback control system to rapidly correct for unexpected changes in ongoing speech (Burnett et al., 1998; Larson & Robin, 2016). In these studies, participants vocalize into a microphone while their auditory feedback is unexpectedly and briefly perturbed in an acoustic dimension such as pitch or formant frequency (Bauer & Larson, 2003; Burnett et al., 1998; Purcell & Munhall, 2006). When feedback is unexpectedly perturbed, speakers rapidly produce a reflexive response (Behroozmand et al., 2012; Burnett et al., 1998; Hain et al., 2000; Kim & Larson, 2019; Larson & Robin, 2016; Scheerer & Jones, 2018). This response is thought to be reflexive because of the automatic and involuntary nature of the response and the inability for speakers to suppress it (Bauer & Larson, 2003; Burnett et al., 1998; Zarate & Zatorre, 2008). The magnitude and timing of the response has been measured as an indication of the accuracy of the auditory feedback control system to correct for errors in ongoing speech (Larson & Robin, 2016).

Auditory feedback control in ataxia has been investigated in three recent studies in which participants were asked to hold a sustained-vowel sound for multiple seconds while either pitch or vowel formant frequency was briefly and unexpectedly perturbed through headphone auditory feedback (Houde et al., 2019; Li et al., 2019; Parrell et al., 2017). In all three studies, individuals with ataxia produced significantly larger reflexive responses than the control participants, indicating that the cerebellum over-corrects due to inaccurate error estimation and correction. In this current study, our goal was to further investigate the cerebellar role in auditory feedback control by studying how cerebellar damage disrupts the task-dependent role of auditory feedback control for efficiency.

### b. The role of the cerebellum in task-dependent auditory feedback control

Past research has demonstrated that the sensitivity of auditory feedback control to correct for perceived errors varies by vocal task, likely a tool to maintain efficiency (Chen et al., 2007; Hilger et al., 2022; Hilger et al., 2023; Natke et al., 2003). Reflexive pitch-shift responses are larger in magnitude in sentence production (Chen et al., 2007) and in singing (Natke et al., 2003) than in sustained-vowel production. Additionally, semantic focus also modulates the pitch-shift response, in which sentences with corrective focus elicit larger responses than new information focus (Hilger et al., 2023). This task-dependent nature of auditory feedback control reflects task-based variation in the precision and scale of the auditory target for production efficiency.

Guenther (2016) describes how the size of the auditory target is scaled by the speaking task. For example, target regions for speech sounds shrink when speakers are asked to speak more clearly, resulting in more precise articulation (Perkell et al., 2002). Guenther (2016) attributes this variable target region to a strategy employed by the speech motor system called an *economy of effort:* speakers minimize the amount of movement required for production while maintaining intelligibility for the listener (Lindblom, 1983, 1990). Essentially, speakers tune their production in relation to the speaking context and their judgment of the listener’s ability to access the information in the speech signal (Lindblom, 1990). For example, speakers may adjust their speech differently if they are speaking with a close friend compared to a stranger, or if they are giving a presentation compared to talking in a more casual setting. By scaling the size of the target region for production, the speech motor system can retain efficiency while maintaining intelligibility.

The economy of effort strategy used by the speech motor system helps explain the task-dependent nature of the auditory feedback control system. Larger pitch-shift responses are observed in tasks such as singing and producing a sentence than for simply holding a vowel sound because the auditory target regions for voice *f*_*o*_ are smaller and more precise in singing and sentence-production. Singing requires matching pitch to a musical note and speaking requires the production of intonational patterns for pitch. Therefore, both production tasks require a high level of precision and less room for error. Mismatches between the auditory feedback and the auditory target will be on average greater in these tasks because the auditory target is smaller. On the contrary, sustained-vowel production requires less precision in *f*_*o*_ so detected mismatches will on average be smaller because there is more room for error (i.e., a larger region for the *f*_*o*_ auditory target).

In relation to ataxia, hypermetric responses have been observed for sustained-vowel production when compared with matched control speakers (Houde et al., 2019; Li et al., 2019; Parrell et al., 2017). This finding indicates that the cerebellar impairment results in over-correction in the feedback control system. In the present study, we are interested in investigating the task-dependent role of auditory feedback control in ataxia. Because the cerebellum is important for adjusting the auditory target for the gestural state and the communicative context, we hypothesized that reflexive responses would show less task-dependency in ataxia because the cerebellum is less accurate in adjusting the auditory targets for the speaking context based on sensory information. Therefore, we predicted (i) that speakers with ataxia would generate larger reflexive responses than the healthy control participants in both a sustained-vowel and sentence-production tasks, and (ii) a smaller differential response would be observed between these tasks in the ataxia group. Additionally, we predicted (iii) that sentence focus would not mediate the magnitude of the reflexive response in ataxia as was observed in the healthy speakers in Hilger et al. (2023). These findings would demonstrate a reduced efficiency and accuracy of the auditory feedback control system due to cerebellar damage, reinforcing the important role of the cerebellum for auditory feedback control.

## Methods

### a. Participants with ataxia

This study analyzed data from the same participants in Hilger et al. (2022) and Hilger et al. (2023). Twenty-seven participants with ataxia (9 male, 18 female) were recruited for this study. Ages ranged from 24–79 years (M = 54.3, SD = 15.1). Education ranged from 12–22 years (M = 15.3; SD = 2.5). All participants were native speakers of American English. Participants had normal, or corrected to normal, visual acuity. Out of the twenty-seven participants with ataxia, five participants were excluded from participating in the auditory feedback perturbation tasks in this study: two participants did not pass the pure-tone audiometric thresholds of 40dB or better in both ears at 500, 1000, 2000, and 4000 Hz; two participants exhibited severe dysarthric impairments and were not able to complete the tasks; and one participant had self-reported cognitive difficulty. The remaining 22 participants all passed the hearing and cognitive screenings.

Ataxia diagnosis was confirmed through participant self-reports of neurology and/or genetic testing. Participants were recruited through local support groups, outpatient clinics of local medical/rehabilitation facilities, flyers in the monthly National Ataxia Foundation newsletter (NAF, 2016), social media, word of mouth, the Communication Research Registry at Northwestern University, and the CoRDS registry (Trudeau, 2013; Coordination of Rare Diseases at Sanford). Summary characteristics of speakers with ataxia are provided in Table 1.

Dysarthria type and severity were assessed using the Frenchay Dysarthria Assessment (Enderby & Palmer, 2008), a standardized assessment sensitive to various severity and subtypes of dysarthria. The FDA-2 assesses level of function for speech subsystems, including respiration, articulation, phonation, resonance, and intelligibility. Dysarthria severity was assessed by comparing the level of function across the speech subsystems. This study was approved by the Northwestern University Institutional Review Board.

### b. Healthy control speakers.

Twenty-nine adults, with no reported history of speech, language, or neurological impairment, were recruited for this study as age- and sex-matched control participants (10 males, 19 females). All participants were native speakers of American English. Ages ranged from 24–79 years (M = 54.1, SD = 15.0). Years of education ranged from 12–22 years (M = 17.3; SD = 2.1). Participants had normal, or corrected to normal, visual acuity. Participants passed hearing and cognitive screenings.

### c. Experiment Overview

Transportation was limited for many of the study participants with ataxia, so participants were provided with four options for testing sites: (1) the Speech Physiology Lab at Northwestern University, (2) the Neurology Clinic at the Northwestern Memorial Hospital, (3) a rented office space in Downtown Chicago, or (4) in a quiet room in their home. The experimental tasks consisted of three parts. First, participants were assessed using the FDA-2. Then, speech samples were collected which included a variety of speaking tasks such as passage reading, spontaneous speech, picture description, maximum phonation time, and diadochokinetic rates. Last, participants completed two auditory feedback perturbation paradigm tasks which are described in detail in the next section. For the current study, only the auditory feedback tasks were analyzed.

### d. Auditory Feedback Perturbation Paradigm Tasks

To investigate voice pitch auditory feedback control in ataxia, we conducted a production study to elicit both sustained-vowels and sentences in individuals with ataxia and matched control participants. In the sustained-vowel task, participants were instructed to repeatedly hold an / / sound for three seconds at a time. For the sentence-production task, a visual world paradigm was used to elicit new information and corrective semantic focus patterns. We used a repeated-measures within- and between-subjects design with four independent variables: *group* (ataxia or control), *task* (vowel or sentence), *semantic focus* (new information or corrective), and *perturbation direction* (+/− 200 cents). Two dependent variables were analyzed, pitch-shift reflex magnitude (cents) and peak latency (milliseconds).

### e. Instrumentation

A similar experimental setup was used to conduct the auditory feedback perturbation paradigm as in previous studies (Burnett et al., 1998; Chen et al., 2013; Kim & Larson, 2019; Liu & Larson, 2007). Participants wore Etymotic Insert Earphones (model ER2–14A) and vocalized into an over-ear microphone (AKG, model C420) positioned approximately one inch from the corner of the mouth. The microphone signal was digitized with a MOTU Ultralite mk3 and controlled by MIDI software (Max MSP 7.0, CueMix FX) to present normal and perturbed auditory feedback to the participant (Quadravox, Eventide). For both the sustained-vowel and the sentence-production tasks, brief 200 ms (millisecond) pitch perturbations of +200 cents (100 cents = one semitone), −200 cents, or 0 cents (control trials) of the voice *f*_*o*_ were presented over the headphones in real time with approximately a 12 ms delay due to instrumentation processing speeds.

In order to mask the participant’s bone-conducted feedback, a gain of about 10 dB SPL (Decibel Sound Pressure Level) was applied to the headphone auditory feedback of the participant’s voice resulting in an auditory feedback of around 80–85 dB SPL (Aphex Headpod 4). Recordings of the microphone signal, auditory feedback, and timing pulses to mark the pitch perturbation onset were obtained using a multichannel recording system (AD Instruments, model ML785, PowerLab A/D converter) and LabChart software (AD Instruments, v.7.0) with a sampling rate of 20 kHz. Recordings of speech output and timing pulses were then time-aligned in LabChart software for offline analysis. The timing pulses were used to differentiate pitch perturbation direction for acoustic analyses.

### f. Design and Stimuli

For both the sustained-vowel and the sentence-production tasks, participants followed instructions from a computer monitor and were told to vocalize at a comfortable but stable pitch and loudness level. The sustained-vowel task was completed first. At the start of each trial, words appeared on the screen instructing participants to “Say ‘ahh.’” Participants vocalized the / / sound until an instruction appeared to “Stop.” There was a two second inter-trial rest period before the instruction for the next trial appeared. Participants were informed before the start of the experiment that they could rest between trials for as long as they needed if they did not trigger the microphone by vocalizing. Each trial was triggered by voice onset with an intensity of 70 dB or greater. The onset of the vocalization was detected using a voice onset detector module in MIDI software. The output from the voice onset detector was used to trigger an Eventide Eclipse Harmonizer (Quadravox, Eventide) to produce two pitch perturbations of pseudo-randomized magnitude (+200 cents, −200 cents, 0 cents) at random intervals during the vocalization period. The pitch-perturbed stimuli were delivered with 700–900 ms variable interstimulus intervals within each 3-sec vocalization period. The pitch perturbations had durations of 200 ms in order to elicit the reflexive response that occurs after the pitch perturbation rather than a volitional response that is triggered by longer pitch perturbation stimuli (Burnett et al., 1998; Hain et al., 2000). When a pitch perturbation was presented, the *f*_*o*_ value produced by the participant was shifted +/− 200 cents for the entirety of the 200-msec perturbation region. Participants completed two blocks of 45 trials each for the sustained-vowel task with a total of 90 vocalizations collected for each participant for this task.

For the sentence-production task, participants produced instructions within a visual world paradigm modeled from Ouyang & Kaiser (2015). The results of this task for the control participants were published in Hilger et al. (2023) and are included in the current study to compare with the speakers with ataxia. The same paradigm was included in the current study to compare with the speakers with ataxia and using the same procedures as in Hilger et al. (2023). For details on the paradigm, please refer to Hilger et al. (2023). Participants were told to imagine that they were playing a game with the computer (i.e., a computer-player), which was using the participant’s verbal instructions to move the pictures on the screen accordingly. However, they were told that the computer-player would occasionally make mistakes and move the wrong picture. Color pictures were presented on the screen in circular frames with the picture names displayed underneath each picture.

**Figure 1:**
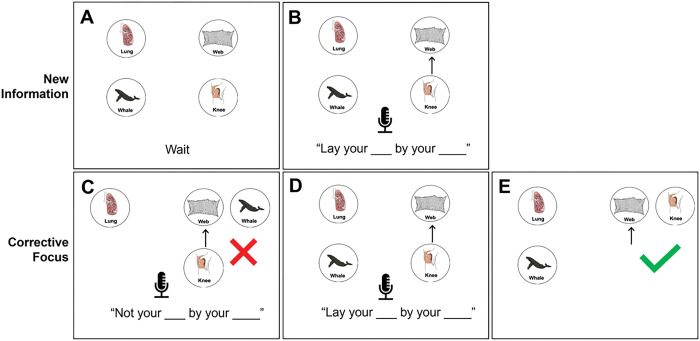
Sample display of the task. The first screen presented is 1A in which four pictures are presented with a cue to wait. In 1B, an arrow appears between *knee* and *net*, cueing the participant to produce the instruction (new focus), “Lay your **knee** by your **net.**” In 1C, an incorrect picture is moved, and the participant is cued to provide a corrective statement, “Not your **WHALE** by your **net.**” In 1D, the participant is cued to repeat the original instruction with corrective emphasis, “Lay your **KNEE** by your **net.**” In 1E, the correct picture is moved.

Based on how the pictures moved and how the computer player responded, the participants were cued to produce either new information focus or corrective focus on the target word, which was the moved object in the visual display. Figure 1B represents the production of a new information focus pattern. The pictures presented on the screen within Figures 1A and 1B are new within the discourse context because they are within a new set of pictures. Therefore, when the instruction is produced, the name of the picture within the instruction cannot be inferred from the previous discourse context. Figures 1C and 1D both represent productions using corrective focus.

The carrier phrase used in this task was, “Lay/not your OBJECT by your LOCATION.” This phrase was chosen because voicing is continuous across the production of the phrase (apart from the break in voicing for the /b/ sound in “by” and the /t/ sound in “not”). Continuous or near-continous voicing was essential to implement pitch perturbations within the phrase and to measure a pitch-shift response, both of which use pitch tracking analyses that require modal voicing. The target word that was manipulated in this task was always the word in the OBJECT position. Words in the OBJECT position occur in the middle of the phrase where modal voicing is frequently used. On the contrary, words in the LOCATION position occur at the end of the phrase, which is a position highly vulnerable to creaky voice (Kreiman, 1982). By manipulating words in the OBJECT position, we hoped to elicit new information and corrective focus for our target words with modal voicing. Target words were chosen from the MultiPic pictures that were monosyllabic and contained all voiced sounds. Participants produced a total of 250 instructive phrases that were subdivided into five blocks of 50 trials each. Within each block, there were around 20 trials each of new information and corrective phrases, depending on the ordering of the pictures. Around 10 trials per block were produced with target words that were neither new information nor bearing corrective focus, but which referred to items that were accessible from the prior context, having been explicitly mentioned in the prior instruction.

**Figure 2:**
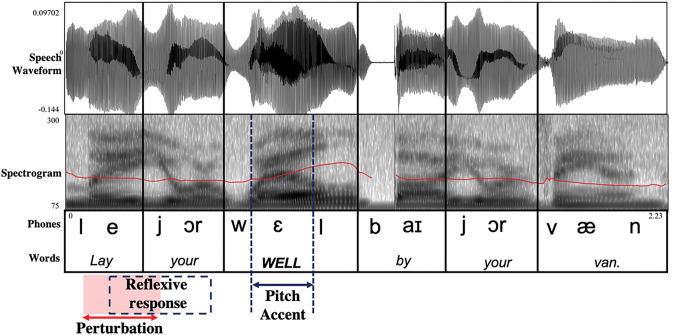
Example timing of the pitch perturbation in the phrase, “Lay your well by your van.” The speech waveform (top) and the spectrogram (middle) are segmented by words and phonemes (bottom). The pitch track is displayed as a red line within the spectrogram. In this example, the target word, *well*, is the stressed word, termed “pitch accent.” The pitch perturbation occurs on the word *lay*, indicated by the red box and arrow at the bottom, and the pitch-shift response occurs shortly after the onset of the perturbation, indicated by the dashed box.

To study the effect of semantic focus on pitch auditory feedback control, brief pitch perturbations were applied in random trials of sentence production. The same perturbation magnitude as the sustained-vowel task was used in the sentence-production task, +/− 200 cents. Pitch perturbations were applied 50 ms after voice onset on the first word in the phrase (i.e., *lay* or *not*) on random trials. Perturbations were 200 ms in duration before auditory feedback was switched back to normal (i.e., unperturbed). We chose to apply the perturbation on the first word in the phrase for two reasons: (1) there is evidence that auditory feedback control is more sensitive at the start of the phrase, possibly because the acoustic features at the start of the phrase are used as a reference to calibrate the relative acoustic production of the rest of the phrase (Hilger et al., 2020; Liu et al., 2009), and (2) we were interested in how auditory feedback control is utilized at the start of the phrase to prepare for anticipatory phrasal stress for semantic focus. This second goal was the topic of a future analysis and was not addressed in this current paper. Figure 2 displays an example production of the phrase, “Lay your **well** by your van” with *well* as the target word. Both the pitch perturbation and the pitch-shift response occur well before the onset of the stressed word (i.e., the pitch accent). At least thirty trials of each perturbation condition (i.e., +200 cents, −200 cents, and 0 cents) were included for each sentence focus type (i.e., new vs. corrective focus), which has been shown to be sufficient for the signal averaging technique used in the pitch-shift response analysis (Bauer & Larson, 2003).

### g. Acoustic Analysis

Acoustic data from the voice recordings for each trial were first analyzed using autocorrelation-based pitch tracking in Praat software to transform the raw data into time-course measures of pitch (Praat Version 6.0.28; Boersma & Weenink, 2019). The sentence productions were then automatically segmented into individual words and phones using the Montreal Forced Aligner (McAuliffe et al., 2017). Segmentation accuracy was confirmed by a final visual inspection. The recorded timing pulses for perturbation onset were aligned with the segmented audio files to label the onset and direction of the pitch perturbation within the production of each phrase. Trials were excluded if the onset of the pitch perturbation did not fall during the production of the first word in the phrase. Trials were also excluded that contained pitch tracking errors, hesitancy, disfluency, or mis-timings in the onset of the pitch perturbations. These exclusion criteria resulted in 25% of the trials being removed, the majority of which (approximately 90%) were due to mis-timings of the pitch perturbation, with the remaining 10% due to pitch tracking errors, hesitancy, or disfluency.

Measurements of the pitch-shift responses (PSR) were performed using Praat software. The voice *f*_*o*_ contours were epoched into segments from 50ms before the perturbation onset (the baseline section) to 400ms after the perturbation onset (post-perturbation window). The voice *f*_*o*_ contours in Hertz were extracted using Praat software and converted to the cent scale using the following formula: Cents = 1200(Log_2_(f2/f1)) where f1equals the mean *f*_*o*_ of the baseline section and f2 equals the mean *f*_*o*_ of the post-perturbation window.

To isolate the PSR from the pitch movement due to phrasal intonation, we completed a difference wave analysis. Without the difference wave analysis, we would not be able to determine if a change in pitch was due to the pitch perturbation or from natural changes in intonation. The difference wave analysis was accomplished by subtracting out the average intonation contour per participant and focus pattern from each individual experimental trial. First, the control trials per participant per focus condition were averaged together to calculate the average intonation contour each participant produced. Then, the average intonation contour was subtracted from the individual experimental trials (i.e., trials with perturbations) for that participant and focus condition. The resulting pitch contours reflected changes in pitch from the pitch perturbation. By completing this analysis for each individual trial, we were able to subtract out variability in intonation that may occur trial-by-trial. This analysis technique has been successfully utilized to analyze the PSR in phrase production for a variety of intonation patterns (Chen et al., 2007; Patel et al., 2019).

The resulting difference waves were then sorted by response direction, i.e., whether the response opposed or followed the direction of the pitch perturbation. Response direction was calculated by comparing the mean *f*_*o*_ of the 50-ms window before perturbation onset with the mean *f*_*o*_ of the 400-ms window after perturbation onset (Behroozmand & Larson, 2011). If the direction of the response and the direction of the perturbation matched (e.g., up–up or down–down), the trial was labeled as “following”; if they differed (e.g., up–down or down–up), the trial was labeled as “opposing.” We decided to include response direction in this analysis because it is not currently well-understood why speakers occasionally follow the perturbation instead of correcting (i.e., opposing) the unexpected change in pitch (Behroozmand et al., 2012; Franken et al., 2018). It is possible that cerebellar disruption influences the direction of the corrective pitch response and could inform our knowledge of following responses in auditory feedback control. Therefore, we included both opposing and following responses in our analyses.

After the voice contours were sorted by perturbation direction and response direction, an event-related averaging was completed by participant that reduced the noise in the audio signal and allowed for extraction of the response (Bauer & Larson, 2003) Essentially, the individual trials were grouped by participant, perturbation direction, and response direction, and then averaged together to compute a final averaged waveform. Response magnitude was then calculated by finding the maximal point (for upward responses) or the minimal point (for downward responses) in a window 60ms-300ms post perturbation-onset. This analysis window was chosen to identify the response magnitude because the minimum latency of the pitch-shift reflex is approximately 60ms after perturbation-onset, according to the timing of muscular activation and corresponding changes in *f*_*o*_ (Kempster et al., 1988; Larson et al., 1987; Perlman & Alipour-Haghighi, 1988), and to avoid capturing a later volitional response that may occur in the 300–400ms window (Hain et al., 2000). Response latency was defined as the time-point of the peak (i.e., the maximal or minimal point) of the PSR.

### h. Statistical Analyses

All statistical analyses were performed using RStudio (Version 1.2.5033) running R Software (Version 3.6.2; R Core Team, 2015) to perform mixed-effects models with the “lme” function from the R package “nlme” (Pinheiro, J., Bates, D., DebRoy, S., Sarkar, D., Heisterkamp, S., Van Willigen, B., & Maintainer, 2017). Mixed-effects models were chosen because of their ability to efficiently handle random by-participant variation (Guilley et al., 1999).

The objective of the first analysis was to separately assess whether the magnitude and peak latency of the pitch-shift response were predicted by group (ataxia vs. control), task (sustained-vowel or sentence-production), sentence focus (new information vs. corrective), perturbation direction (+/− 200 cents), and response direction (opposing vs. following the perturbation direction) while controlling for by-participant variance. After checking for and meeting assumptions of normality, separate models for response magnitude and peak latency were run. For each model, response magnitude and peak latency were separately used as dependent variables. In the first model, group, task, perturbation direction, and response direction were used as fixed effects, and participant was included as a random effect. In the second model, only the sentence-production task was analyzed to determine the effect of sentence focus on response magnitude and latency by group. Significant effects from the mixed-effects models were assessed by pairwise comparisons of Least Squares Means from the R package “lsmeans” (Lenth, 2017). Cohen’s D was calculated for each significant effect using the “lme.dscore” function from the R package “EMAtools” (Kleiman, 2017).

A final exploratory analysis was performed to compare the number of opposing and following responses by group, task, perturbation direction, and sentence focus pattern. Response direction was converted to a binary variable and used as a dependent variable in a logistic regression with group, task, perturbation direction, and sentence focuses included as fixed effects using the “glm” function in R.

## Results

### a. Production of Phrasal Stress by Sentence Focus

**Figure 3:**
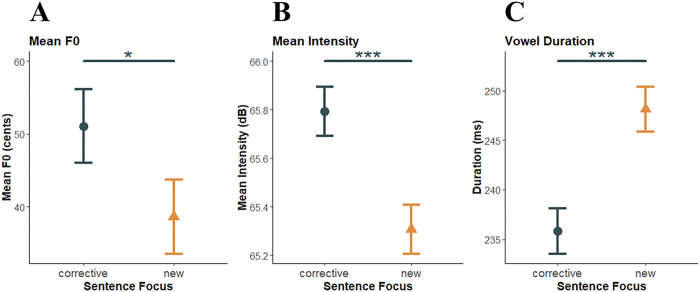
Production of the stressed word by sentence focus for the ataxia group. 3A displays the mean and standard error for mean *f*_*o*_ of the stressed word for corrective focus (black circle) and new focus (gold triangle). 3B displays the same mean and standard error for mean intensity, and 3C for vowel duration. Statistical significance is indicated by stars corresponding to p-values (* *p* < 0.05; ** *p* < 0.01; *** *p* < .001).

The motivation for this first analysis was to determine whether the production task successfully elicited corrective and new focus in individuals with ataxia. In Hilger et al. (2023), we observed that the control group increased mean *f*_*o*_ and mean intensity of the stressed word for corrective focus compared to new focus (however the opposite effect was observed for vowel duration). Figure 3 displays the means and standard errors for mean *f*_*o*_ (cents), mean intensity (dB), and duration (ms) of the stressed word by sentence focus for the ataxia group for the control trials only (i.e., the trials without perturbations). Significant effects were found for mean *f*_*o*_ (*F*(1, 2721) = 5.14, *p* = .02, d = 0.09), mean intensity (*F*(1, 3059) = 37.20, *p* < .0001, d = 0.22), and duration (*F*(1, 3059) = 26.20, *p* < .0001, d = −0.29). Mean *f*_*o*_ of the stressed word was significantly higher for corrective focus (*M* = 51.07 cents, *SE* = 5.05) than for new focus (*M* = 38.65 cents, *SE* = 5.12). Mean intensity was also significantly higher for corrective focus (*M* = 65.79 dB, *SE* = 0.10) than for new focus (*M* = 65.31 dB, *SE* = 0.10). A different trend was observed for vowel duration that was significantly increased for new focus (*M* =248.15 msec, *SE* = 2.28) than for corrective focus (*M* = 235.84 msec, *SE* = 2.31). Overall, mean *f*_*o*_ and mean intensity were increased for corrective focus, but duration was increased for new focus. These findings match the patterns observed in the control participants in Hilger et al. (2023).

### b. Pitch-shift Response Magnitude by Task and Group

**Figure 4:**
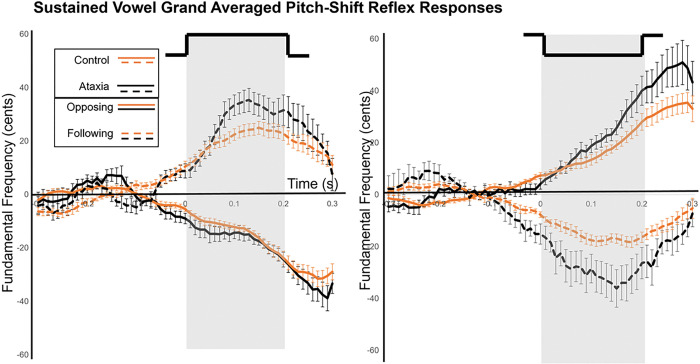
Sustained-vowel grand averaged pitch-shift responses for all participants by upward perturbations (left-hand column) and downward perturbations (right-hand column). The duration of the perturbation is indicated by the gray bar. Solid lines indicate opposing responses (top row) and dashed lines indicate following responses (bottom row). The ataxia group is indicated by the black lines and the control group is indicated by the gold lines. Error bars represent standard error.

**Figure 5:**
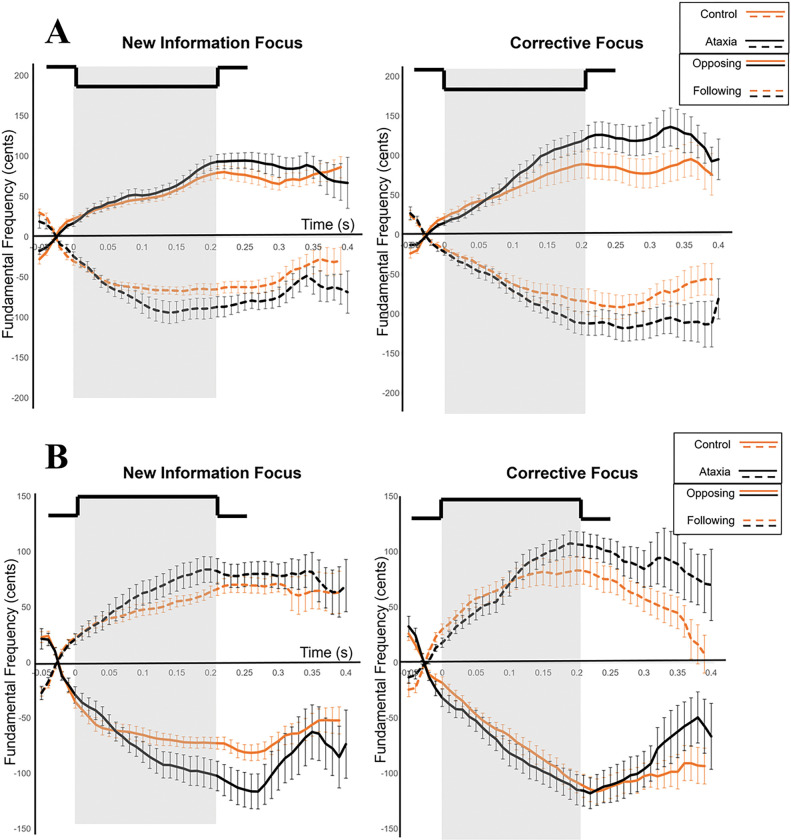
Sentence-production grand averaged pitch-shift responses for all participants by downward perturbations (5A) and upward perturbations (5B). The duration of the perturbation is indicated by the grey bar. Solid lines indicate opposing responses and dashed lines indicate following responses. The ataxia group is represented by the black lines and the control group is indicated by the gold lines. New information focus is in the left-hand columns for both figures and corrective focus is the right-hand columns. Error bars represent standard error.

Figure 4 displays the grand averages of the voice *f*_*o*_ responses in sustained-vowel production to upward and downward pitch perturbation stimuli for participants with ataxia and control participants. Figures 5A and 5B display the grand averages of the voice *f*_*o*_ responses in sentence-production to downward (5A) and upward (5B) pitch perturbation stimuli for new information and corrective focus and for participants with ataxia and control participants. The first analyses compared response magnitude by experimental condition. A significant main effect was shown for task, *F*(1, 662) = 424.64, *p* < .0001, d = 1.11. Across both participant groups, pitch-shift response magnitudes were significantly larger in sentence-production (*M* = 113.39 cents, *SE* = 2.39) than in sustained-vowel production (*M* = 37.94 cents, *SE* = 1.74). A second main effect was observed for response direction, *F*(1, 662) = 8.39, *p* = .004, d = 0.23. Across both tasks and groups, larger response magnitudes were measured for opposing responses (*M* = 97.78 cents, *SE* = 3.10) than for following responses (*M* = 90.32 cents, *SE* = 3.10). Finally, a third main effect was observed for group, *F*(1, 47) = 4.10, *p* = .05, d = 0.66. Across both tasks, larger response magnitudes were measured for the ataxia group (*M* = 102.36 cents, *SE* = 3.85) than for the control group (*M* = 88.22 cents, *SE* = 2.56). Refer to Table 2 for pitch-shift response magnitudes by task and group. Overall, response magnitudes were significantly larger in phrase production, for opposing responses, and for the ataxia group.

### c. Pitch-shift Response Magnitude by Sentence Focus and Group

**Figure 6:**
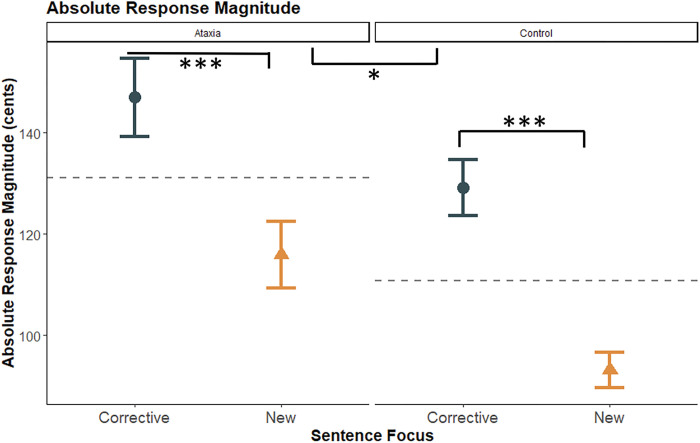
Participant group by sentence focus for absolute response magnitude. The ataxia group is displayed in the left-hand column with the control group displayed in the right-hand column. Corrective focus is displayed in black and new information focus is displayed in gold. Group averages are indicated by a dashed line in each panel. Statistical significance for between-group comparisons is indicated by stars corresponding to p-values (* *p* < 0.05; ** *p* < 0.01; *** *p* < .001). Comparisons are indicated by black brackets positioned between two points.

A second mixed-effects model was run to compare the effect of sentence focus on absolute response magnitude in the phrase production task. Two main effects were significant: sentence focus and group. For both groups, the pitch-shift response magnitudes were significantly larger with corrective focus (*M* = 136.71 cents, *SE* = 4.61), than new information focus (*M* = 102.81 cents, *SE* = 3.52) (*F*(1, 328) = 51.32, *p* < .0001, d = 0.40) (Figure 6). Additionally, the ataxia group had larger responses across both focus patterns than the control group (Ataxia: *M* = 123.24 cents, *SE* = 4.23; Control: *M* = 106.41 cents, *SE* = 2.70) (*F*(1, 47) = 3.97, *p* =0.05, d = 0.48). No other main effects or interactions were significant. Even though the ataxia group had larger response magnitudes than the control group for both corrective focus (Ataxia: *M* = 146.98 cents, *SE* = 7.74; Control: *M* = 129.89 cents, *SE* = 5.53) and new focus (Ataxia: *M* = 115.89 cents, *SE* = 6.53; Control: *M* = 93.12 cents, *SE* = 3.50), the interaction of group by focus was not significant (*p* = 0.57). Overall, these results show that though both groups produced significantly larger pitch-shift response magnitudes in corrective focus than in new focus, the ataxia group had significantly larger responses than the control group.

### d. Peak Response Latency

For peak response latency, pitch-shift responses occurred on average 216.44 msec (*SE* = 7.53) after perturbation onset for the ataxia group, and on average 209.91 msec (*SE* = 5.03) for the control group. Although responses occurred earlier in the control group, this difference was not statistically significant (*F*(1, 47) = 0.78, *p* = 0.38, d = 0.13). A significant main effect was observed for response direction *F*(1, 662) = 39.37, *p* < .0001, d = 0.49. Across groups and tasks, following responses were significantly faster (*M* = 195.18 msec, *SE* = 4.04), than opposing responses (*M* = 229.37 msec, *SE* = 3.65).

### e. Response Direction

The final analysis was exploratory to compare the number of opposing and following responses by group, task, perturbation direction, and sentence focus pattern to determine if an experimental condition elicited more opposing or following responses. Additionally, we asked the exploratory question of whether cerebellar disease would elicit more following responses, potentially due to inaccurate estimation in the direction of the error and/or the direction of the correction. There was a total of 204 potential averaged responses for the sustained-vowel production task (51 participants X 2 perturbation directions X 2 response directions) and 408 averaged responses for the phrase-production task (51 participants X 2 perturbation directions X 2 response directions X 2 sentence focus patterns). After analysis, 566 averaged responses were measured out of a total of 612 potential responses. Table 3 lists the number of responses by experimental condition. Not all participants opposed and followed every perturbation direction for each experimental condition, which accounts for the number of “non-responses” indicated in the table. There were no significant differences in the count of responses for any condition (*p* > 0.05), indicating that both groups of speakers opposed and followed the perturbation for all perturbation direction, task, and sentence focus conditions.

## Discussion

The purpose of this study was to investigate the task-dependent role of pitch auditory feedback control in ataxia to measure the effect of cerebellar damage on the efficiency and accuracy of the auditory feedback control system. Impaired auditory feedback control has been measured in a number of studies on cerebellar damage, showing hypermetric responses in simple sustained-vowel tasks (Houde et al., 2019; Li et al., 2019; Parrell et al., 2017). These results demonstrate inaccuracy of the cerebellum in estimating the amount of correction needed in response to detected changes in auditory feedback. In this study, we were interested if cerebellar damage would also affect the efficiency of auditory feedback control for scaling auditory targets appropriately for the speaking context. According to Lindblom (1983), the speech motor control system employs a strategy of economy of effort in order to reduce the magnitude and velocity of articulator movement required in speech production while maintaining intelligible speech. This strategy can be accomplished by scaling the size of the auditory target for production so that the system retains efficiency while maintaining intelligibility. Previous research has shown that healthy speakers produce larger pitch-shift responses in more complex tasks, such as sentence-production or singing, than for more simple production tasks (Chen et al., 2007; Natke et al., 2003). For example, people produce large responses in sentences with more salient semantic focus patterns, such as corrective focus compared to new information focus (Hilger et al., 2023). We predicted that individuals with ataxia would produce larger responses than the control group, similar to prior research, but that they would not have differentially larger responses in more complex tasks. In other words, we predicted large responses for the ataxia group across all tasks, regardless of task or focus pattern. This result would reflect cerebellar damage for accuracy of auditory feedback correction (i.e., responses are too large) as well as efficiency of correction (i.e., responses are not scaled by vocal task).

We found that, overall, the ataxia group produced larger pitch-shift responses than the control group for both the sustained-vowel and the sentence-production tasks. These results support the findings from previous studies showing that ataxia results in hypermetric compensatory responses to unexpected changes in auditory feedback. From the current study, over-corrected responses were observed across speaking tasks for both simple tasks (i.e., sustained-vowel production) and more complex tasks (i.e., sentence-production) in ataxia. This over-corrected response demonstrates the inaccuracy of the feedback control system for appropriately scaling the corrective movement for error.

Next, we assessed the efficiency of auditory feedback control in ataxia by measuring whether task-dependent scaling of auditory targets was retained. Contrary to our hypothesis, we observed a task-dependency in auditory feedback control in ataxia, finding that participants with ataxia were able to produce a differential response magnitude both by task and by semantic focus pattern. Similar to the control speakers, speakers with ataxia produced larger pitch-shift responses in the sentence-production task than the sustained-vowel task, and for sentences with corrective focus than for new information focus. Overall, this finding demonstrates that despite the cerebellar disruption, the motor speech system is still able to employ the economy of effort strategy by scaling the auditory target by speaking context. For example, the larger responses observed in the sentence-production task for both groups demonstrates that the auditory target was scaled down for precision in this task, resulting in a greater detected mismatch. It is possible that efficiency is retained in the speech motor control system in ataxia to scale the auditory target by speaking task, even though accuracy is reduced from cerebellar damage when correcting for errors. Another interpretation is that attentional demands for sentence production as well as for corrective focus could influence feedback correction, resulting in larger responses. This interpretation is not mutually exclusive with the theory of economy of effort and also aligns with the neural mechanisms studied in this paper. Higher-level cortical mechanisms are typically preserved in ataxia, which are the mechanisms responsible for attention. In this case, these higher-level cortical mechanisms may be responsible for scaling the auditory target based on attentional demands as well as economy of effort.

These findings have implications for both our understanding of the role of the cerebellum in feedback control as well as our clinical approach to treating speech naturalness in ataxic dysarthria. For the role of the cerebellum, individuals with cerebellar damage exhibit a hypermetric response to auditory feedback perturbations across speaking tasks, but the task-dependent role of auditory feedback control remains intact. This demonstrates that the auditory target was effectively scaled to the demands of the speaking task, despite the inaccuracy of the corrective response. A potential explanation for this finding is that there may be several variables that are used to scale the auditory target, some of which might involve more cortical processes rather than cerebellar processes. For example, scaling the target may include factors such as the speaking context, the current gestural state, task complexity, prosodic targets, and fatigue. Any combination of these factors may scale the production target greater or smaller in size depending on whether greater precision is required for the speech production task. Since we found that the pitch-shift response was effectively scaled by production task and semantic focus pattern in ataxia, it is likely that there are higher-level cortical processes that are involved with scaling production of targets for these factors. Overall, these findings show that cerebellar damage disrupts the accuracy in estimating corrective responses to unexpected auditory feedback perturbations, but efficiency for scaling the response by task and sentence focus remains intact.

Clinical techniques can take advantage of the intact task-dependency in auditory feedback control in cerebellar patients by addressing the inaccurate, over-correction in pitch control. As found in Hilger et al. (2022), speech naturalness is more significantly impaired in ataxic dysarthria than speech intelligibility, reflecting the patterns in disrupted prosody. The prosodic impairments are likely in-part due to over-correction in *f*_*o*_, and probably intensity, in online speech production. Because the original auditory target is inaccurately estimated by cerebellar processes, more errors may be detected, triggering more corrective movements, which are also likely to be inaccurately estimated. We theorize that this feedback impairment results in a pattern of frequent and over-estimated corrections in pitch, and likely loudness as well. This impairment would help explain the variable pitch and loudness characteristics observed in ataxic dysarthria. To apply this work clinically, a treatment study could test the use of other feedback mechanisms, such as visual and kinesthetic feedback, to improve prosodic control by strengthening the internal models of the auditory targets. Since the task-dependent role of auditory feedback is intact, the production target itself is not wholly compromised in ataxia and has potential for responding to rehabilitative techniques.

Another factor we analyzed in this study was response direction, or whether the speaker opposed or followed the direction of the perturbation. Similar to the results from the control speakers in Hilger et al. (2023), there were no significant differences in the number of opposing or following responses for any task or group in this study. A potential hypothesis was that the cerebellar damage in ataxia would result in more following responses because the estimated corrective movement is inaccurate. However, this hypothesis was not supported because the speakers with ataxia opposed and followed the perturbations to the same extent as the control speakers. A simple explanation for when the response follows the perturbation is that it is possible that the direction of the response occurs due to chance.

Limitations in this study include both the nature of the task as well as the number of perturbation variables that were analyzed. Although we attempted to elicit more natural productions using a visual-world paradigm, the task still involved a rather unnatural-sounding carrier phrase because of the constraint for voiced sounds with the words in the phrase. Additionally, the sentence-production task did not involve the same level of complexity as spontaneous speech or conversation entails, which may demonstrate interesting findings in relation to task complexity. Lastly, only one pitch perturbation magnitude was tested in this study because of an attempt to limit the number of trials needed. However, because we only studied one perturbation magnitude, we could not measure a differential response to different perturbation magnitudes in the production tasks. Next steps in this research should include various perturbation magnitudes and various tasks of differing complexities. Despite these limitations, clear effects were measured by group and task that shed light on the role of the cerebellum in auditory feedback control.

## Conclusion

The purpose of this study was to measure the task-dependent role of auditory feedback control in individuals with ataxia to investigate how accuracy and efficiency are affected by cerebellar damage. We found that the ataxia group produced larger pitch-shift responses than the control group for both the sustained-vowel and sentence-production tasks. Additionally, a differential response magnitude was observed by task and by semantic focus pattern in both the ataxia group and the control group. These findings demonstrate that even though accuracy is affected by cerebellar damage, as evidenced by the hypermetric responses, the system still retains efficiency in utilizing the task-dependent role of auditory feedback.

## Figures and Tables

**Table 1:** Participant Characteristics. Participants are listed by group (AT = ataxia, CO = control), participant number, sex (M = male, F = female), education, ataxia diagnosis (SCA = spinocerebellar ataxia, AOA = ataxia with oculomotor apraxia, SCAR = spinocerebellar ataxia recessive autosomal, FA = Friedreich's Ataxia), disease duration, and dysarthria severity.

Participant Group	Participant Number	Sex	Age	Education (years)	Ataxia Diagnosis	Disease Duration	Dysarthria Severity
AT	1	M	67	14	SCA-Unknown	2.5	Mild
AT	2	M	47	14	SCA-Unknown	23	Mild-Moderate
AT	3	M	72	22	SCA6	3	Severe
AT	4	F	62	14	SCA6	1	Mild-Moderate
AT	5	F	42	16	SCA2	0.5	Mild-Moderate
AT	6	M	36	12	SCA7	0.5	Mild
AT	7	M	55	14	SCA1	22	Severe
AT	8	M	24	14	SCA2	3	Mild
AT	9	F	67	16	SCA6	20	Mild-Moderate
AT	10	F	41	18	SCA3	10	Mild-Moderate
AT	11	F	55	14	SCA3	0.5	Mild
AT	12	F	63	14	SCA6	3	Mild
AT	13	F	69	15	SCA-Unknown	10	Moderate
AT	14	F	70	16	SCA3	5	Mild
AT	15	M	64	12	SCA15	24	Mild
AT	16	F	65	14	SCA-Unknown	7	Mild-Moderate
AT	17	F	62	18	Gluten Ataxia	14	Mild
AT	18	F	36	18	SCA5	13	Mild
AT	19	F	42	18	AOA2	23	Mild-Moderate
AT	20	F	60	18	SCAR8	21	Mild-Moderate
AT	21	M	55	16	FA	14	Mild-Moderate
AT	22	F	76	14	SCA6	9	Mild-Moderate
AT	23	F	55	18	SCA-Unknown	2	Moderate
AT	24	F	79	12	SCA-Unknown	3	Mild
AT	25	M	31	12	FA	0.5	Mild-Moderate
AT	26	F	47	18	SCA-Unknown	25	Mild-Moderate
AT	27	F	28	12	FA	12	Mild-Moderate
AT = Ataxia, CO = Control, SCA = Spinocerebellar Ataxia, FA = Friedreich's Ataxia, M = Male, F = Female							
CO	1	M	68	18			
CO	2	M	45	16			
CO	3	M	71	18			
CO	4	F	61	12			
CO	5	F	38	18			
CO	6	M	38	18			
CO	7	M	55	18			
CO	8	M	24	16			
CO	9	F	65	16			
CO	10	F	40	16			
CO	11	F	51	12			
CO	12	F	66	18			
CO	13	F	70	22			
CO	14	F	70	18			
CO	15	M	63	18			
CO	16	F	63	18			
CO	17	F	60	18			
CO	18	F	36	22			
CO	19	F	41	18			
CO	20	F	58	18			
CO	21	M	50	18			
CO	22	F	71	16			
CO	23	F	79	16			
CO	24	F	54	18			
CO	25	M	36	18			
CO	26	F	42	18			
CO	27	F	23	20			
CO	28	F	62	18			
CO	29	M	70	18			

**Table 2:** Absolute response magnitude by task and group. Task is listed in the left-hand column, followed by group, mean response magnitude, and standard error.

Task	Group	Mean	Standard Error
Sustained-Vowel	Control	34.69 cents	1.62
	Ataxia	42.70 cents	3.43
Phrase	Control	106.41 cents	2.70
	Ataxia	123.24 cents	4.23

**Table 3:** The number of opposing and following responses by task and group. Response direction (opposing and following) is listed on the left-hand column, followed by task and group. Non-responses are listed at the bottom of the table.

Response Direction	Task	Group	Number of Responses
Opposing	Vowel	Ataxia	42
		Control	55
	Phrase	Ataxia	82
		Control	111
Following	Vowel	Ataxia	35
		Control	51
	Phrase	Ataxia	81
		Control	109
Non-Responses	Vowel	Ataxia	11
		Control	10
	Phrase	Ataxia	13
		Control	12

## Data Availability

Data are available at https://osf.io/cu5wy/
